# Scarlet Fever

**Published:** 1857-04

**Authors:** Reuben Searcy

**Affiliations:** Tuscaloosa, Ala.


### ARTICLE II.

*Scarlet Fever.* By REUBEN SEARCY, M. D., Tuscaloosa, Ala.

In 1853, Scarlet Fever broke out in the county and city of Tuscaloosa, as an epidemic, destroying many children. I encountered a number of cases, and lost, probably, about one-third of my patients—nearly all of the malignant ones. At that time, the more violent the attack, the more active was the treatment. Bleeding, emetics, active cathartics, blistering the neck, &c., were resorted to. The disease and the treatment, both prostrating the patient, often brought him to a speedy end.

The theory, then, was, that the more inflammatory the attack was, it must be met by a corresponding depletory and antiphlogistic treatment. The theory was very plausible; but it carried death in its sequel.

We consulted authorities, and we consulted physicians abroad: we could obtain no better light. After a while, we saw that a milder course of treatment succeeded better. We soon abandoned blistering over the throat and glands of the neck, when we had the mortification of witnessing gangrene and sloughing ensuing.

We now have adopted a theory and practice, obtained from reading and observation, that has proved so successful, that we deem it expedient to give them publicity.

The disease may be atmospherical, dropping down in a community without our knowing whence it comes; but we know that it is contagious, and very probably, that in those places where it is supposed to break out atmospherically, it may have been communicated by a grown person, who has contracted the disease, and carried it into a neighborhood, he having only the Scarlet fever sore throat, without knowing it, and supposing he had a very sore throat from a bad cold. In this way, I have known it introduced into families by adults.

May 29th, 1855. I visited a young man just returned from Mobile—he having a very sore throat and some fever, which lasted some four or five days, and he convalesced. I supposed at the time, as he had been very much exposed night and day on the river, that his attack was attributable to his exposure, and as he had no eruption on his skin, I did not suspect Scarlet fever. He was sick in a well ventilated upstairs room, and his two grown sisters were his nurses: all the children were kept away, because he could not bear any noise on account of great pain in his head. About twelve or fourteen days after, the two sisters were taken with decided Scarlet fever, and from these two cases, some of the children down stairs contracted the disease in ten or twelve days after, and it went through the family. Again. In December, 1856, Mr. C. M. F., who resides out of the city, had a servant girl at work in the city. She was taken with a very sore throat, and went home, and was treated for quinsey by her mistress, and ten or twelve days after, the whole family of children in the same room, were taken with Scarlet fever. I could mention other cases, if necessary, of adults having only the sore throat, and going about mixing with the people, and diffusing the disease with their poisonous breath. In this way it may be introduced into communities, without their knowing the disease was near them, and who knows but that it is propagated from place to place in this way, when it is supposed to originate spontaneously?

I believe the principal seat of the disease is in the Tonsils, and that the eruption is one of the marked signs of the disease. You can see the Tonsils enlarged two or three days before there is any development of fever, and throwing the child's head back, you will discover that the enlarged Tonsils will show externally, near the angle of the jaws, in an elongated swelling of two or three inches in length on both sides, which is not apt to be the case in colds.

In the beginning of the attack, if the bowels are constipated, I give a small dose of castor oil. If they are in a natural condition, I give no aperient. I endeavor to keep up a diaphore sis, with equal parts of sweet spirits of Nitre and Paregoric and an eighth of Cox's hive syrup mixed, and given: To a child one year old, ten or twelve drops; and five years old, half of a tea-spoon full, every two or three hours, and if the child becomes drowsy from the effects of the paregoric, give less or at longer intervals, and let the child drink some sage or balm teas, not very strong. Externally, have the patient greased from head to foot, very often, with a fat piece of bacon. The lard in the bacon keeps the skin soft, and the salt that is in solution in the grease, proves sufficiently stimulating to the capillaries, to excite perspiration. The scarf skin that is parched and burnt by the fever, and killed by the eruption, does not let the perspiration sufficiently through the pores, unless softened by some kind of oil. The rubbing on of the grease has a very quieting effect. I have a very fat piece of bacon about half of an inch thick, and as wide as the neck is long, tacked on to a piece of cloth and worn as a cravat night and day, renewing it once a day.

I cauterized the tonsils, when very sore or ulcerated, with a solution of the nitrate of silver, of from ten to forty grains, according to the age of the person, about twice a day. I wish my patients to drink very often of corn meal gruel made thin and seasoned only with a little salt. They may eat mush and molasses, bread, crackers, &c., and drink tea and coffee, but no milk, except the child, its mother's milk. I wish to keep and sustain all the strength I can, in my patients. If the throat becomes so swollen that the child cannot swallow, sustain it with gruel injections, administered every five or six hours. I sustained a little girl three days in this way, the swelling then subsiding, she swallowed and got well.

The disease may be rendered entirely harmless, if a proper hygienic course be observed. When a case is introduced into a family, put every child and young person, and mother who has a child at the breast, upon a strickly vegetable diet, as much so as if you were wishing to prepare them for the inoculation of the Small pox. Every case that takes the disease when thus prepared, will be mild, and in many you will only discover a slight swelling of both Tonsils, which is as likely to prevent their having the disease again, as if the eruption was fully out; and when a child has had this swelling, it is no longer necessary to diet more. But if any child has not had the swelling, continue the dieting for two weeks after the last case of Scarlet fever.

I have never seen a bad case when the system was thus prepared, and in my practice, the first case is the one I dread the most. I do not want to weaken any of the children by dieting; let them eat enough of a vegetable and milk diet to keep up their strength.

It is well known to Surgeons and Physicians, that the inflammatory symptoms run much higher in those cases, when the blood is made from meat or grease, than when the system is properly prepared, and as Scarlet fever is highly inflammatory in its character, hence the importance of preparing the system for its advent, in all young persons exposed to the contagion. Old persons rarely have more than the Scarlet fever sore throat.

